# The Oncogenic Role of Tribbles 1 in Hepatocellular Carcinoma Is Mediated by a Feedback Loop Involving microRNA-23a and p53

**DOI:** 10.3389/fphys.2017.00789

**Published:** 2017-11-10

**Authors:** Ying Ye, Guangdong Wang, Guoyu Wang, Juhua Zhuang, Saifei He, Yanan Song, Jing Ni, Wei Xia, Jiening Wang

**Affiliations:** ^1^Department of Nuclear Medicine, The Seventh People's Hospital of Shanghai University of Traditional Chinese Medicine, Shanghai, China; ^2^Department of Science and Technology, Shanghai University of Traditional Chinese Medicine, Shanghai, China

**Keywords:** hepatocellular carcinoma, Tribbles 1, microRNA-23a, tumor suppressor p53, β-catenin signaling pathway

## Abstract

Hepatocellular carcinoma (HCC) is a common malignancy associated with a high risk of recurrence and metastasis and a poor prognosis. Here, we examined the involvement of the pseudokinase Tribbles 1 (TRIB1), a scaffold protein associated with several malignancies, in HCC and investigated the underlying mechanisms. TRIB1 was upregulated in HCC tissues and cell lines in correlation with low levels of p53. TRIB1 gain and loss of function experiments indicated that TRIB1 promoted HCC cell viability concomitant with the downregulation of p53, and induced HCC cell migration, invasion, and epithelial-mesenchymal transition. TRIB1 was identified as a target of microRNA-23a (miR-23a), and miR-23a overexpression downregulated TRIB1 and upregulated p53 in HCC cells. Ectopic expression of TRIB1 upregulated β-catenin and its effectors c-myc and MMP-7 in a p53-dependent manner. TRIB1 silencing inhibited tumor growth and promoted apoptosis *in vivo* via a mechanism that would involve the modulation of p53 and β-catenin signaling. The present results indicate that TRIB1 promotes HCC tumorigenesis and invasiveness via a feedback loop that involves the modulation of its expression by miR-23a with the likely downregulation of p53, and suggest the involvement of the β-catenin signaling pathway. These findings suggest potential targets for the treatment of HCC and therefore merit further investigation.

## Introduction

Hepatocellular carcinoma (HCC), which accounts for 85–90% of all liver cancers, is the second leading cause of cancer-related mortality worldwide (Forner et al., 2012). Surgical resection and liver transplantation are the first choice treatments for HCC in the early stages; however, not all patients are eligible for surgery and the prognosis of patients with HCC remains poor, mainly because of the high rate of recurrence and metastasis (Bosetti et al., 2014). The high degree of vasculature in the liver increases the risk of intrahepatic and extrahepatic metastasis, contributing to treatment failure (Wang Z. et al., 2013).

Epithelial mesenchymal transition (EMT), by which polarized epithelial cells acquire a mesenchymal phenotype characterized by fibroblastoid-like shape, high motility, and increased invasive capacity, plays a critical role in the invasion and metastasis of cancer cells (Kalluri and Weinberg, 2009). The transforming growth factor beta (TGF-β) and wnt/β-catenin signaling pathways play important roles in EMT. β-catenin, which plays an important role in cell adhesion, forms a complex with E-cadherin and is involved in the maintenance of cell-cell contact (MacDonald et al., 2009). β-catenin accumulates in the nucleus and binds to the transcription factor TCF/LEF, modulating the transcription of genes involved in EMT (Gilles et al., 2003; Yook et al., 2006).

The pseudokinase Tribbles 1 (TRIB1) has a conserved motif similar to the catalytic domain of a serine/threonine kinase but lacks an ATP binding or kinase catalytic domain (Hegedus et al., 2006; Yokoyama and Nakamura, 2011). TRIB1 acts as a scaffold or adaptor protein, promoting the degradation of target proteins, and regulating several important signaling pathways (Cunard, 2013). TRIB1 is overexpressed in AML and myelodysplastic syndrome and in certain solid tumors, including prostate, thyroid, ovarian, and colorectal cancers (Puskas et al., 2005; Puiffe et al., 2007; Liang et al., 2013; Lin et al., 2014; Mashima et al., 2014). Likely as a result of physical interactions, TRIB1 inhibits the tumor suppressor p53, which is the most frequently mutated gene in HCC and plays a critical role in many cancers (Miyajima et al., 2015). p53 functions as a transcriptional factor, activating genes involved in cell-cycle progression, senescence, and apoptosis (Kruse and Gu, 2009).

MicroRNAs (miRNAs) are small non-coding RNAs of 20–22 nucleotides that modulate gene expression by binding to the 3′-untranslated region (3′-UTR) of target mRNAs (Pillai et al., 2007). Aberrant expression of miRNAs is associated with the pathogenesis of several diseases including cancer, and miRNAs can act as tumor suppressors or oncogenes depending on their specific targets (Gu et al., 2012; Nair et al., 2012). miR-23a upregulation is associated with the growth and invasiveness of HCC cells and related to poor prognosis in HCC (Bao et al., 2014). miR-23a was found to be associated with the chemosensitivity of HCC cells through the modulation of its target topoisomerase 1, and the expression of miR-23a is positively regulated by p53 (Wang N. et al., 2013).

In the present study, the upregulation of TRIB1 in HCC tissues and cell lines led us to investigate the involvement of TRIB1 in HCC. We discovered a regulatory network involving miR-23a, p53, and TRIB1 and elucidated a potential mechanism underlying the invasiveness of HCC cells through the modulation of β-catenin signaling. Our results identify potential biomarkers in HCC, as well as possible therapeutic targets for the treatment of liver cancer.

## Materials and methods

### HCC tissue samples and cell culture

HCC and adjacent normal tissues were obtained from patients in Seventh People's Hospital of Shanghai. Informed consent was obtained from each patient, and the study was approved by Institute Research Ethics Committee of the Seventh People's Hospital of Shanghai University of TCM.

Human HCC (HepG2, SMMC-7721 and Huh7 cells) and the normal liver cell line LO2 were obtained from the Cell Bank at Chinese Academy of Sciences (Shanghai, China) and cultured in Dulbecco's modified Eagle's medium (DMEM, HyClone, USA) supplemented with penicillin (100 IU/mL), streptomycin (100 mg/mL) and 10% (vol/vol) heat-inactivated fetal bovine serum (Gibco, USA) at 37°C in a 5% CO2 incubator.

### Immunohistochemistry and immunofluorescence

Tissues were dehydrated and embedded in paraffin. Sections were dewaxed in xylene and rehydrated in an alcohol series. Antigen retrieval was achieved by incubation in citric acid buffer for 10 min. After blocking in 3% hydrogen peroxide and 5% bovine serum albumin, sections were incubated overnight at 4°C in anti-TRIB1 antibody (1:500, Santa Cruz, CA, USA). Sections were then rinsed in PBS, incubated with secondary antibodies for 30 min at room temperature, stained with diaminobenzidine tetrahydrochloride and counterstained with hematoxylin for 5 min. Slides were observed under a microscope and photographed.HepG2 cells were resuspended in culture medium and seeded at densities of 2 × 10^4^ cells/well in 24-well Transwell inserts (8 μm pores, CorningCostar, Rochester, NY, USA) then incubated for 24 h at 37°C. After 24 h, cells were treated with 3.7% formaldehyde in phosphate-buffered saline at room temperature for 20 min. After washing with phosphate-buffered saline, coverslips were incubated in blocking buffer [1% bovine serum albumin in tris-buffered saline tween-20 (TBST)] for 1 h. Cells were subjected to immunostaining with p53 antibody (1: 200 dilution) or TRIB1 antibody (1: 200 dilution) for 1 h and washed three times with TBST, followed by staining with Alexa Fluor 488 (1:200 dilution in blocking buffer) or Alexa Fluor 594 for 1 h. After washing at least three times with TBST, the coverslips were mounted using commercial mounting medium (Kirkegaard and Perry laboratories, Gaithersburg, MD) and were examined by immunofluorescence microscopy with Hamamatsu digital camera using a 60 × oil immersion objective and MetaVue software.

### Plasmids and siRNA

Expression vectors encoding the TRIB1 or p53 genes, and small interfering RNA (siRNA) against TRIB1 or p53 were purchased from Shanghai GenePharma Co. Ltd. (Shanghai, China). HepG2 and Huh7 cells (5 × 10^5^ cells) were cultured in 6-well plates with antibiotic-free DMEM for 24 h and then transfected with the indicated siRNAs and vectors using Lipofectamine 2,000 (Invitrogen, USA) according to the manufacturer's protocol. Knockdown efficiency was determined by qRT-PCR and western blot analysis.

### MTT assays

Cell viability was assessed using the MTT assay. Cells were seeded at a density of 1,000 per well in 96-well plates and incubated for the indicated time points, followed by addition of 20 μL of 5 mg/mL MTT (Sigma, USA) and incubation at 37°C for 3 h. Then, 250 ml DMSO was added and absorbance was read at 490 nm using a microplate reader (Bio-Rad).

### RNA isolation and quantitative real-time PCR

The knockdown or overexpression efficiency was determined by quantitative real time PCR at 24 h after transfection. Total RNA was extracted using the Trizol reagent (Invitrogen, USA).Real-time quantitative PCR (qRT-PCR) was performed using a SYBR green qPCR SuperMix-UDG kit (Life Technologies, USA) on an ABI PRISM 7300 system (Applied Biosystems). Ct values of mRNA were normalized to β-actin. Relative expression was calculated using the ΔΔCt method.

### Western blot analysis

Total proteins were extracted with RIPA buffer and protein concentration was determined using the BCA protein assay kit (Thermo). Approximately 30 μg of protein from each sample was separated using a 10% SDS-polyacrylamide gel and transferred to PVDF membranes. Membranes were blocked with 5% skim milk in TBST, and incubated in the following primary antibodies overnight at 4°C: p53 (1:2,000); TRIB1(1:2,000); MMP7(1:5,000); β-catenin (1:5,000); c-myc (1:2,000); E-cadherin (1:2,000) and N-cadherin (1:2,000), all purchased from Santa Cruz Biotechnology (Santa Cruz, CA, USA), the catalog number are as follows: p53: sc-47698; TRIB1: sc-431763; MMP7: sc-421677; β-catenin: sc-419477; c-myc: sc-400001; E-cadherin: sc-437341; N-cadherin: sc-419593. After incubation in the corresponding secondary antibodies for 1 h at room temperature and washing in TBST, proteins were detected using Super ECL Plus Detection Reagent (Applygen, Beijing, China).

### Transwell migration and invasion analysis

For migration assays, cells were seeded at a density of 1 × 10^4^ in the upper chamber of Transwell plates in 100 μL of serum-free medium. Complete medium was added to the lower chamber as a chemoattractant. Cells were incubated for 24 h at 37°C, and the cells remaining on the upper surface were removed with a cotton swab. The cells migrated to the lower surface were fixed with 4% paraformaldehyde, stained with crystal violet solution, and photographed and counted. The cell invasion assays were performed using the same method except that 100 μL of Matrigel in DMEM was added to the upper chamber at 6 h before the seeding of cells.

### Luciferase reporter assay

The 3′-UTR sequence of TRIB1 was amplified from human genomic DNA and subcloned into the luciferase reporter vector. HepG2 cells (4 × 10^4^) were seeded in 24-well plates and co-transfected with wild-type (wt) or mutant (mut) 3′-UTR vectors and miR-23a or miR-NC using Lipofectamine 2000. After 48 h, HepG2 cells were assessed for luciferase activity using the Dual Luciferase Reporter Assay System (Promega) according to the manufacturer's protocol. The experiments were performed in triplicate.

### Tumor formation assay in nude mice

Six-week-old male BALB/c nude mice (*n* = 10) were obtained from Shanghai Laboratory Animal Company (SLAC, Shanghai, China). Cultured HepG2 cells of control (shNC) and shTRIB1 were resuspended cells in PBS. Mice were randomly divided into two groups of five mice each, and 6 × 10^6^ cells (shTRIB1 or shNC) in 100 μL of PBS were inoculated subcutaneously into the left armpit of each mouse. After 7 days, tumor diameters were measured with a Vernier caliper every 7 days and tumor volumes were calculated using the following equation: tumor volume = 1/2 (length × width^2^). After 5 weeks, mice were sacrificed and tumors were separated.

### Statistical analysis

Statistical analyses were performed with SPSS 17.0 software. The statistical significance of differences was determined by either the Student's *t*-test for comparison between means or one-way analysis of variance. Data were considered to be statistically significant at ^*^p < 0.05 and ^**^p < 0.01.

## Results

### TRIB1 is overexpressed in human HCC tissues and cell lines

**Figure 1:**
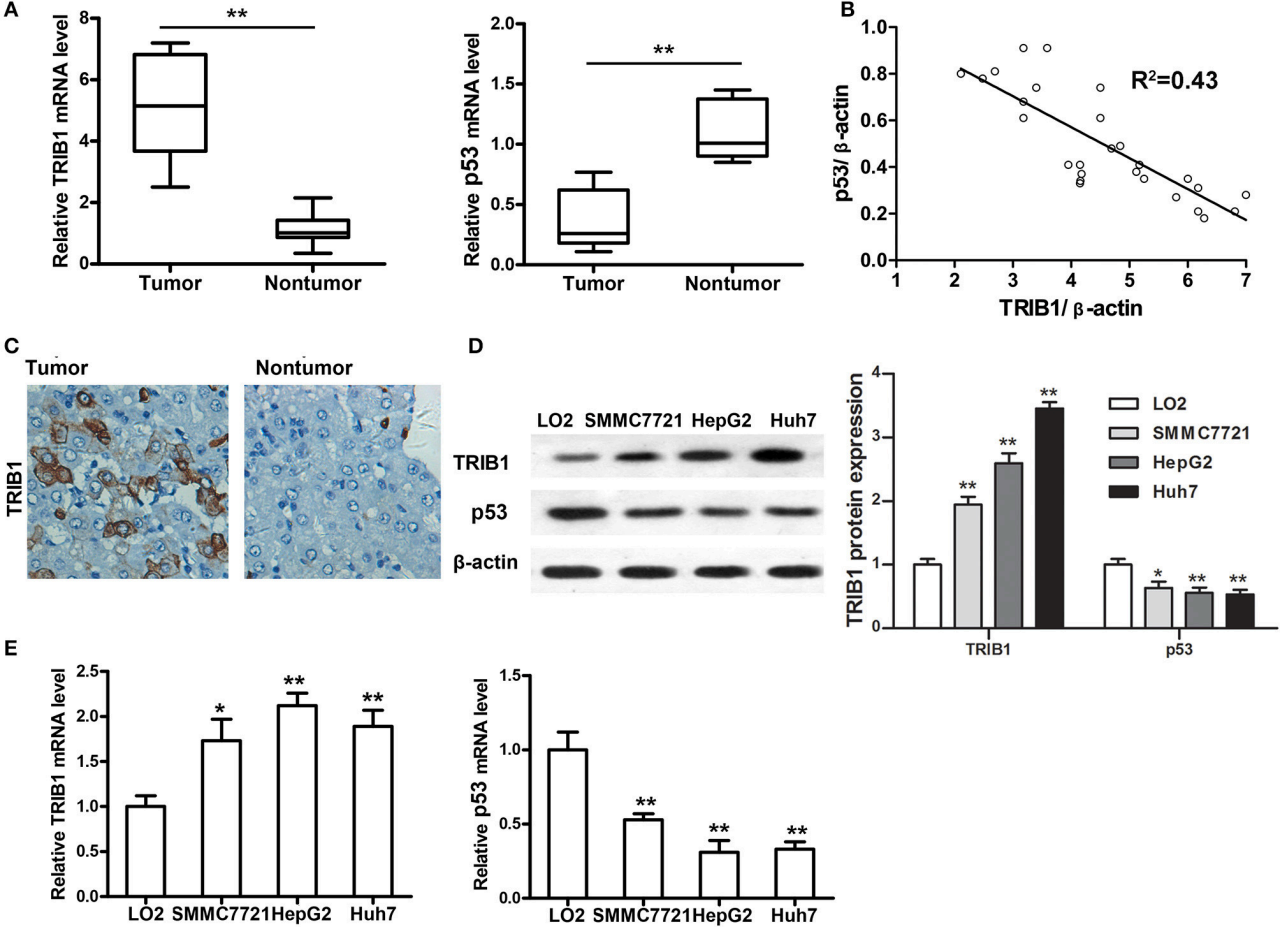
TRIB1 is overexpressed in human HCC tissues and cell lines. **(A)** qRT-PCR analysis of TRIB1 and p53 mRNA expression in HCC tumors and adjacent non-tumor tissues. **(B)** We have made a pooled dot plot to verify the correlation again in HCC patients. **(C)** Immunohistochemical analysis of TRIB1 in HCC (left panel) and adjacent liver tissues (right panel) (original magnification, × 200). **(D)** Western blot analysis of TRIB1 protein expression in the normal liver cell line LO2 and the HCC cell lines SMMC-7721, HepG2, and Huh 7. β-actin was used as an internal control. **(E)** qRT-PCR analysis of TRIB1 and p53 mRNA expression in HCC and normal liver cells. The data were normalized to the expression levels in LO2 cells. Bar graphs (mean ± SEM) and representative images are shown. ^*^p < 0.05, ^**^p < 0.01 compared with the LO2 group (*n* = 3).

The expression of TRIB1 and p53 was measured by qRT-PCR in tumor and adjacent non-tumor tissues from patients with HCC. The results showed that TRIB1 was upregulated and p53 was downregulated in tumor tissues compared with their levels in non-tumor tissues (Figure 1A). We have made a pooled dot plot to verify the correlation (Figure 1B) in HCC patients. The results confirmed that the linear inversely correlation between TRIB1 and p53. These results were confirmed by immunohistochemical staining of HCC and non-tumor tissue sections (Figure 1C). Western blot and qRT-PCR analysis of TRIB1 and p53 expression in the HCC cell lines SMMC-7721(wide type p53), HepG2(wide type p53), and Huh7(mutant p53 Y220C) and the normal liver cell line LO2 showed that TRIB1 expression was significantly higher and p53 expression was significantly lower in HCC cells than in normal liver cells (Figures 1D,E).

### TRIB1 downregulates p53 and promotes HCC cell growth

**Figure 2:**
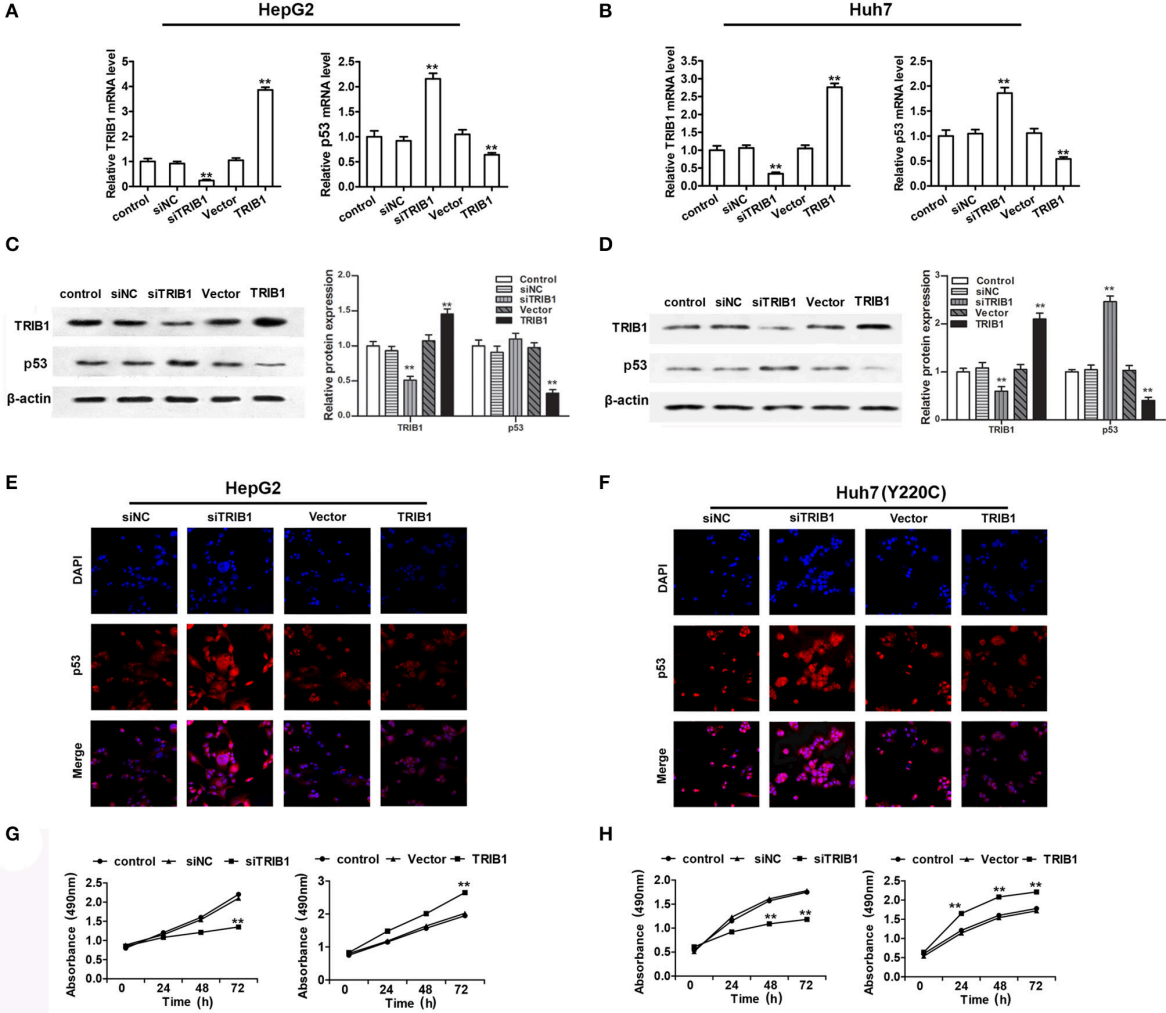
TRIB1 downregulates p53 and promotes HCC cell growth. **(A–D)** The mRNA and protein expression levels of TRIB1 and p53 in HepG2 **(A,C)** and Huh7 **(B,D)** cells treated with or without siTRIB1 or negative control (siNC) or TRIB1 plasmid were determined by qRT-PCR and western blotting. ^*^p < 0.05 or ^**^p < 0.01, compared with the control group. Immunocytochemistry were used to detect the p53 and TRIB1 expression in **(E)** HepG2 cells and **(F)** Huh7 (Y220C) cells. Cell growth of HepG2 **(G)** and Huh7 **(H)** cells treated as described above was determined using the MTT assay at the indicated time points. ^*^p < 0.05 or ^**^p < 0.01, compared with the control group (*n* = 3).

To examine the correlation between TRIB1 and p53 in HCC, HepG2 and Huh7 cells were transfected with siRNA against TRIB1 or control siRNA or a plasmid overexpressing TRIB1, and the expression of TRIB1 and p53 was measured by qRT-PCR, western blotting and immunocytochemistry. The results showed that knockdown of TRIB1 significantly upregulated p53 at the mRNA (Figures 2A,B,E,F) and protein (Figures 2C,D) levels in HepG2 and Huh7 cell lines, whereas TRIB1 overexpression had the opposite effects. The effect of TRIB1 knockdown and overexpression on cell proliferation was examined in HepG2 and Huh7 using the MTT assay. The results showed that TRIB1 silencing significantly inhibited cell proliferation, whereas TRIB1 overexpression significantly increased cell proliferation after 24 h in both HCC cell lines (Figures 2G,H).

### TRIB1 promotes HCC cell migration and invasion and EMT

**Figure 3:**
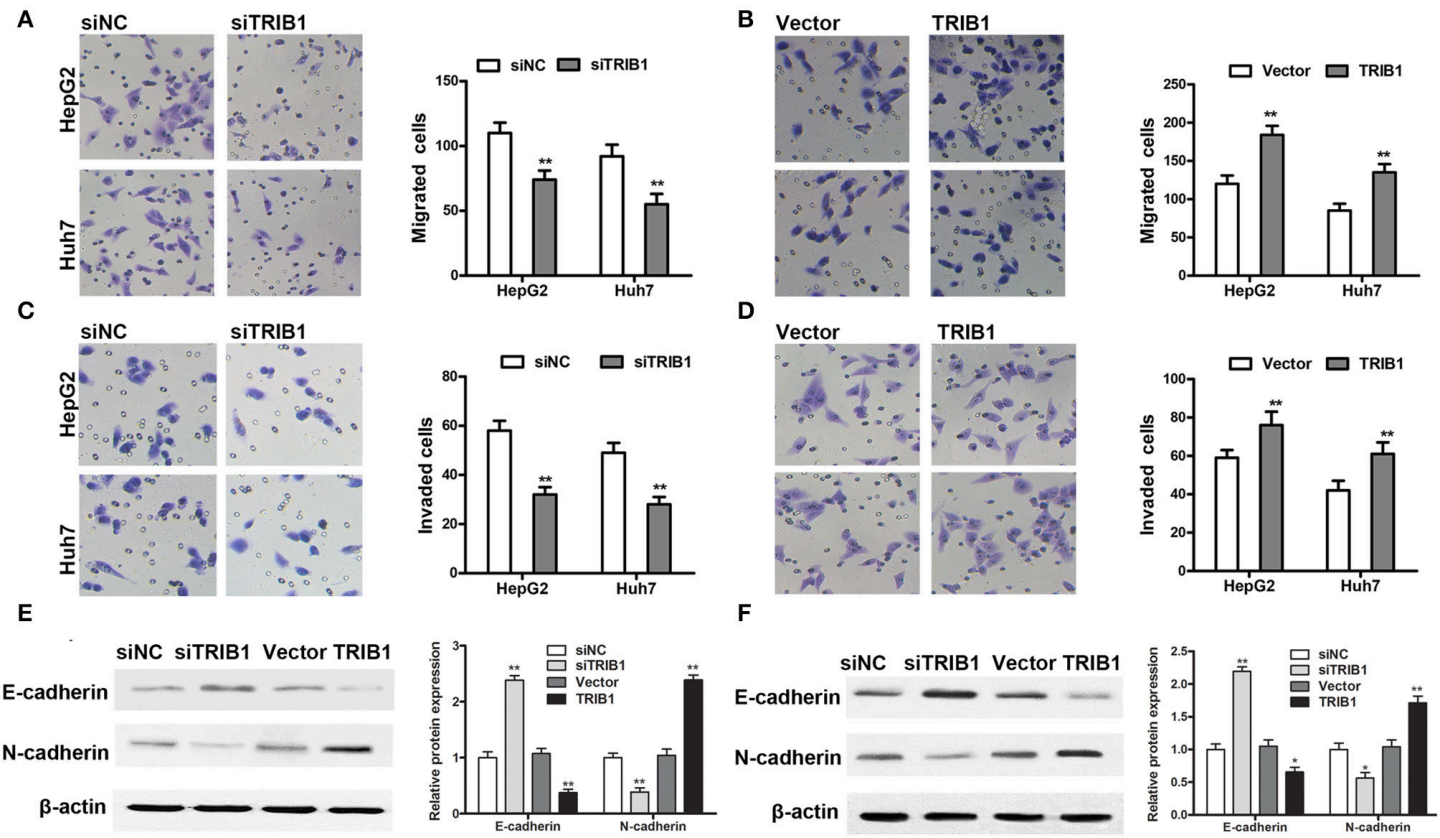
Effects of TRIB1 on the migration/invasion of HCC cells and EMT markers. **(A–D)** The migratory and invasive abilities of HepG2 and Huh7 cells with TRIB1 silencing **(A,C)** or overexpression **(B,D)** were assessed using Transwell assays. **(E,F)** Western blot analysis of the protein levels of E-cadherin and N-cadherin in HepG2 **(E)** and Huh7 **(F)** cells transfected with siNC or siTRIB1 or Vector or TRIB1. The data are representative of at least three independent experiments (*n* = 3). ^*^p < 0.05, ^**^p < 0.01, compared with the siNC or Vector group.

The effects of TRIB1 knockdown and overexpression on HCC cell migration and invasion were examined by Transwell assays in HepG2 and Huh7 cells. The results showed that TRIB1 silencing significantly inhibited the migratory and invasive abilities of both cell lines, whereas TRIB1 overexpression had the opposite effects (Figures 3A–D). Western blot analysis of markers of EMT showed that TRIB1 silencing upregulated the epithelial marker E-cadherin and downregulated the mesenchymal marker N-cadherin, whereas TRIB1 overexpression had the opposite effects (Figures 3E,F). Taken together, these results indicated that TRIB1 promotes cell migration and invasion and induces EMT in HCC.

### miR-23a directly targets TRIB1 and is involved in a feedback loop with the TRIB1/p53 axis

**Figure 4:**
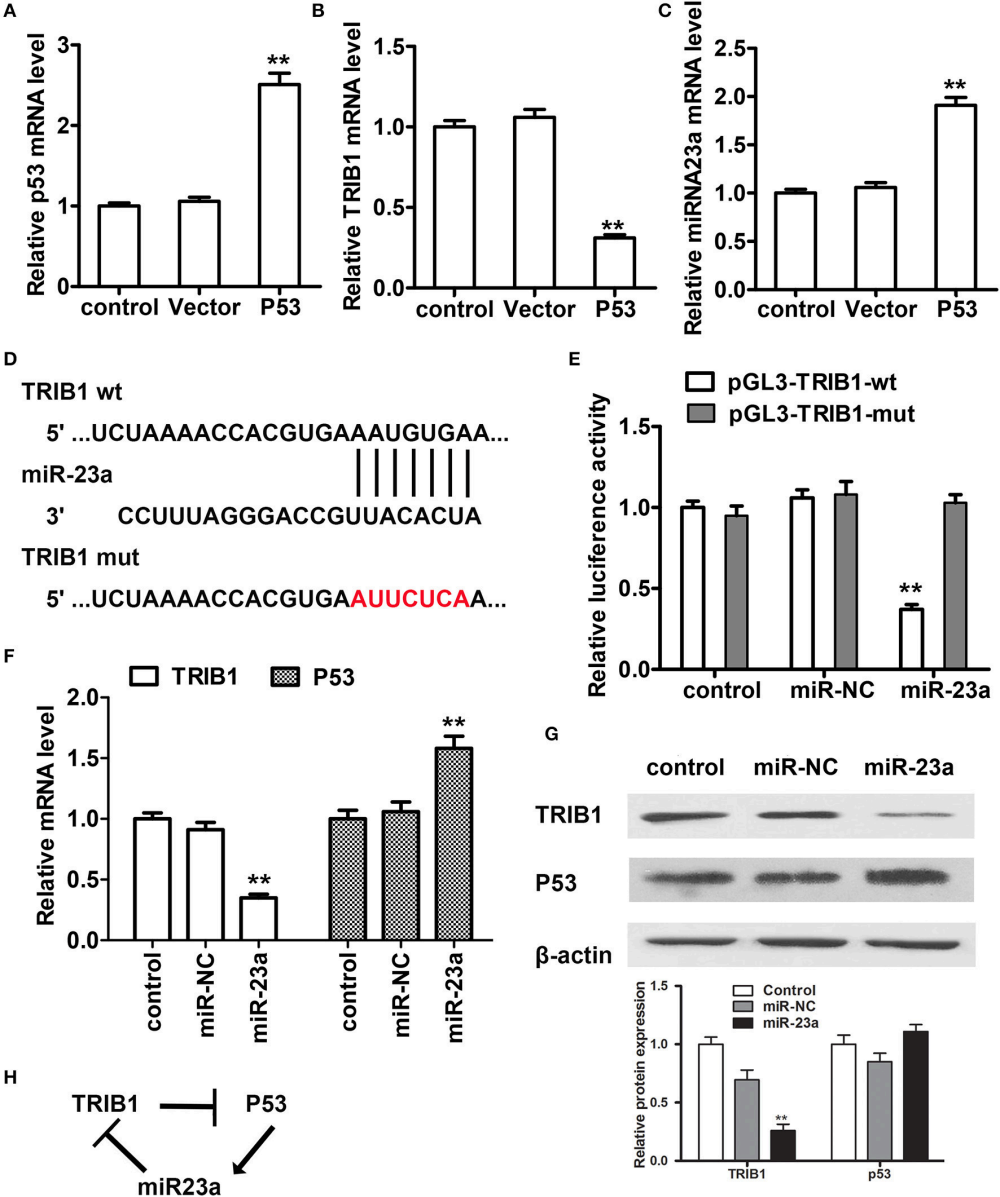
miR-23a regulates the TRIB1/p53 axis in HCC cells. **(A–C)** The expression levels of p53, TRIB1, and miR-23a were examined in HCC cells transfected with or without a p53 overexpression plasmid by qRT-PCR. **(D)** TRIB1 is a predicted target of miR-23a by TargetScan. **(E)** HepG2 cells were co-transfected with miR-23a and wild-type (wt) or mutant (mut) pGL3-TRIB1 constructs and subjected to luciferase reporter assays. **(F,G)** The expression levels of TRIB1 and p53 in HepG2 cells transfected with or without miR-NC or miR-23a were determined by qRT-PCR and western blotting. **(H)** Schematic showing the miR-23a/TRIB1/p53 feed-forward loop (*n* = 3). ^*^p < 0.05, ^**^p < 0.01 compared with the control group.

To further examine the regulation of TRIB1 and p53 in HCC, we searched for a regulatory miRNA involved in the modulation of TRIB1 expression based on algorithm prediction of miRNA binding sites in the 3′-UTR of TRIB1 (Soubeyrand et al., 2016a) and the TargetScan-based prediction of TRIB1 as a target of miR-23a. First, the effects of p53 overexpression were examined in HepG2 cells. The results showed that p53 overexpression significantly downregulated TRIB1 mRNA and significantly upregulated miR-23a (Figures 4A–C). Figure 4D shows the TRIB1 3′-UTR and miR-23a complementary sequences and the TRIB1 mutant 3′-UTR construct. Co-transfection of cells with 3′-UTR reporter constructs and miR-23a and the results of the luciferase reporter assay showed that miR-23a significantly inhibited the wild-type TRIB1 promoter activity, whereas it had no effect on the mutant 3′-UTR reporter activity (Figure 4E). These results suggested that miR-23a directly targets the 3′-UTR of TRIB1. To confirm the role of miR-23a in the regulation of the TRIB1/p53 axis, the expression levels of TRIB1 and p53 were examined by qRT-PCR and western blotting in HepG2 cells. The results showed that miR-23a significantly downregulated TRIB1 and upregulated p53 (Figures 4F,G). Expression of both wild-type and several mutant p53 that we detected in HepG2, including Y220C, K132E, and R249S, all three are prevalent p53 mutant in HCC, greatly increased the level of miR-23a compared with the control vector without altering the level of pri-miR23a (Supplementary Figure 1). Therefore, the elevation of p53 (both wild-type and mutants) could promote the processing of miR-23a. Taken together, these results suggested a feed-forward loop by which miR-23a negatively regulates TRIB1, leading to the upregulation of p53 (both wild-type and mutants) and the induction of miR-23a expression (Figure 4H).

### TRIB1 stimulates β-catenin signaling in a P53-dependent manner

**Figure 5:**
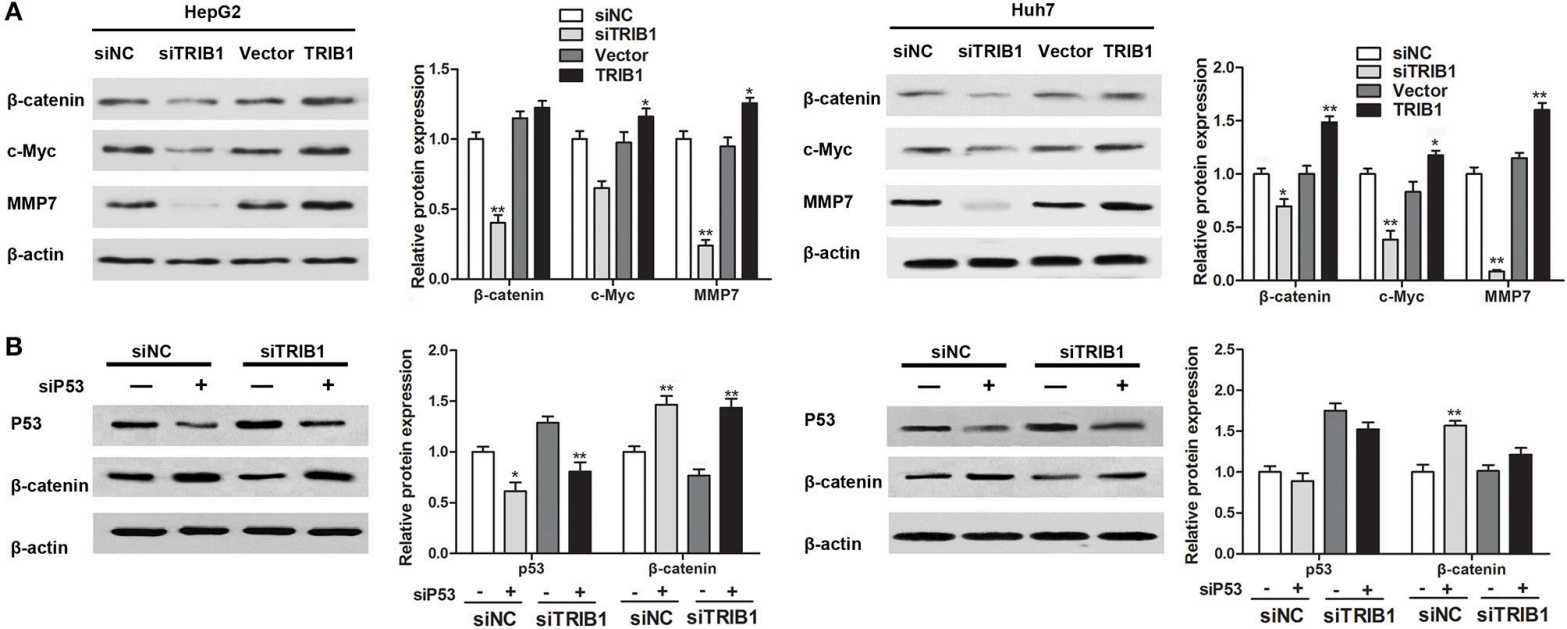
TRIB1 stimulates β-catenin signaling in a p53-dependent manner. **(A)** The expression of β-catenin, c-Myc, and MMP7 was detected by western blotting in HepG2 and Huh-7 cells transfected with siNC or siTRIB1 or Vector or TRIB1. β-actin was used as a loading control. ^*^p < 0.05, ^**^p < 0.01 compared with the siNC group (*n* = 3). **(B)** Effect of p53 and TRIB1 silencing on β-catenin expression in HepG2 and Huh-7 cells was detected by western blotting. ^*^p < 0.05, ^**^p < 0.01 compared with the siNC[siP53(−)] group (*n* = 3).

The effects of the TRIB1/p53 axis on HCC were further examined by assessing the expression of β-catenin and its target genes c-myc and matrix metalloprotease-7 (MMP-7) in HepG2 and Huh7 cells in response to TRIB1 silencing or overexpression. The results of western blot analysis showed that knockdown of TRIB1 downregulated β-catenin, c-myc, and MMP-7 in both HCC cell lines, whereas TRIB1 overexpression had the opposite effect (Figure 5A). Silencing of p53 reversed the effect of TRIB1 knockdown on the downregulation of β-catenin, suggesting that the effect of TRIB1 on β-catenin signaling is dependent on p53 (Figure 5B).

### TRIB1 knockdown inhibits *in vivo* HCC tumor formation via P53

**Figure 6:**
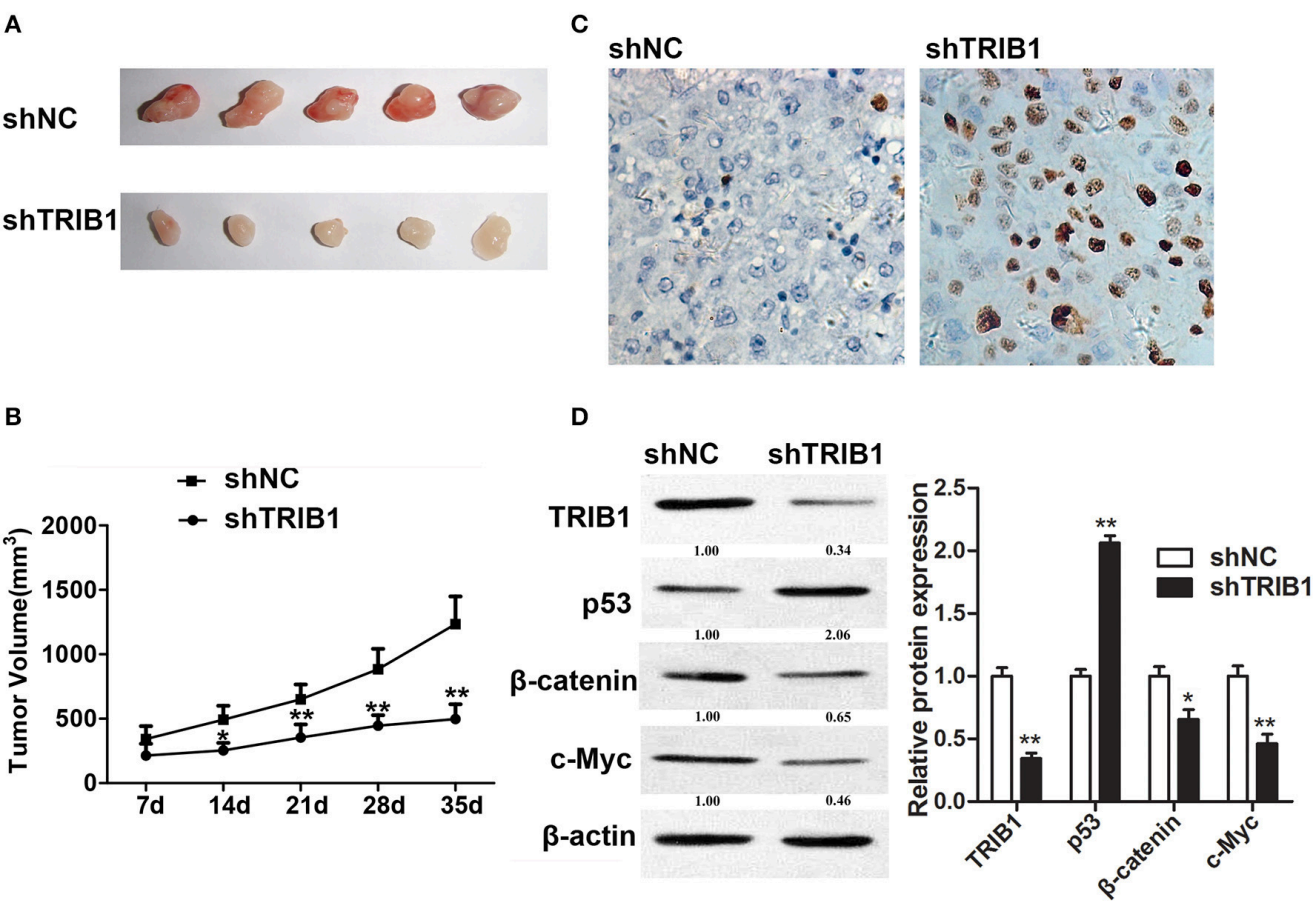
TRIB1 knockdown inhibits *in vivo* tumor formation from HepG2 cells in nude mice. Stable TRIB1 knockdown and control HepG2 cells were implanted subcutaneously in the left flank of nude mice. **(A)** After 35 days, mice were sacrificed and tumors were excised. **(B)** Effect of TRIB1 knockdown on tumor volume in a nude mice model. **(C)** The TUNEL assay (×400) was performed to determine the apoptotic indices. **(D)** Western blot analysis of the expression of p53, β-catenin, and c-myc in tumor tissues from control and shTRIB1 mice. β-actin was used as an internal control. Bar graphs (mean ± SEM) and representative images are shown. ^*^p < 0.05, ^**^p < 0.01 compared with the control group (*n* = 3).

An *in vivo* HCC tumor model was generated by implantation of HepG2 cells with stable TRIB1 knockdown into nude mice. Figure 6A shows representative images of tumors from shNC and shTRIB1 mice. Assessment of tumor volume at the indicated time points showed that TRIB1 silencing significantly inhibited tumor growth (Figure 6B). The results of the TUNEL assay showed an increase in apoptosis in shTRIB1 mice compared to that in shNC mice (Figure 6C). Western blot analysis of tumor tissue lysates showed that p53 expression was higher, whereas β-catenin and c-myc expression was lower in TRIB1 knockdown tumors than in those from shNC mice (Figure 6D). Taken together, these results indicated that TRIB1 knockdown inhibited tumor growth *in vivo*, likely through the induction of p53 mediated apoptosis.

## Discussion

The present study examined the role of miR-23a and its target TRIB1 in HCC and elucidated a potential underlying mechanism that would involve the modulation of p53 and the β-catenin pathway. The role of p53 in the regulation of miR-23a and the inhibition of p53 by TRIB1 were described previously (Miyajima et al., 2015). We identified TRIB1 as a target of miR-23a and showed that TRIB1 is upregulated in HCC tissues and cell lines in correlation with the downregulation of p53. TRIB1 has been implicated in the pathogenesis of various diseases including cancer, and the regulation of TRIB1 stability and levels remains a subject of controversy (Soubeyrand et al., 2016a). Given the importance of TRIB1 in pathological processes, elucidating the mechanisms underlying the modulation of TRIB1 levels is important. The stability of TRIB1 is in part regulated by the proteasome, and the regulation of TRIB1 expression was shown to play an important role in hepatocytes (Soubeyrand et al., 2016b). The role of miRNAs in the regulation of TRIB1 expression was suggested previously in a study showing that miR-224 downregulation in prostate cancer may promote tumor progression and poor outcomes through the upregulation of its target TRIB1 (Lin et al., 2014). The results of the present study suggest that miR-23a modulates TRIB1 expression, and high levels of TRIB1 in HCC may lead to the inhibition of p53 and increased tumorigenesis.

The role of p53 in the regulation of micro-RNA has been reported previously. Knockdown of p53 by si-RNA decreased the level of miR-18a, whereas overexpression of either wild-type or mutant p53 increased the level of miR-18a (Li et al., 2015). The present results indicated that the expression of both wide-type and several mutant p53 that we detected in HCC, including K132E, WY, Y220C, and R249S, increased the level of miR-23a significiently. The results demonstrate that the miR-23a could modulate the expression of TRIB1 and the overexpression of TRIB1 also inhibits the p53 expression (wide-type and mutant p53). In brief, the TRIB1, p53 (both wide-type and mutant: Y220C) and miR-23a partly form a feedback loop for a potential targets for the treatment of HCC have been demonstrated. Other munant p53 (K132E, WY, and R249S) in this feedback loop needs to be investigated in future.

The present results suggested that TRIB1 promoted HCC cell migration and invasion and induced EMT through the upregulation of β-catenin, which led to the upregulation of c-myc and MMP-7. Ectopic expression of TRIB1 promoted HCC cell migration and invasion and induced EMT, and these effects were accompanied by the upregulation of β-catenin. Furthermore, our results suggested that the effects of TRIB1 on β-catenin were p53-dependent, as the effects of TRIB1 knockdown were restored by p53 silencing. The role of the β-catenin/MMP7 axis and p53 in HCC invasiveness and EMT was reported previously (Chen et al., 2013; Wang Z. et al., 2013; Yuan et al., 2014). However, few studies examined the involvement of the TRIB family in HCC or in the regulation of Wnt/β-catenin signaling. TRIB2, which is involved in tumorigenesis in various cancers, was shown to increase β-catenin nuclear accumulation in liver cancer cells (Xu et al., 2014). However, this increase in nuclear β-catenin led to the inhibition of Wnt activity by promoting the association of β-catenin with TRIB2-associated ubiquitin E3 ligases, leading to its destabilization. TRIB2 was shown to integrate Wnt/β-catenin signaling in liver cancer cells, although it was suggested to act downstream of Wnt (Wang J. et al., 2013), which differs from the present results. The specific effect of TRIB1 on Wnt/β-catenin signaling and whether this mechanism could underlie the effect of TRIB1 on promoting metastasis in HCC needs to be investigated further.

TRIB1 negatively regulates the tumor-Suppressor activity of p53, The activity of the tumor-suppressor p53 is regulated through the mechanism of the phosphorylation, acetylation, and methylation (Brooks and Gu, 2003; Olsson et al., 2007). Acetylation of p53 increases its activity and stability, and TRIB1 suppresses p53 activity by promoting its deacetylation (Miyajima et al., 2015). p53 is a positive regulator of miR-23a, and several studies suggested the involvement of a miR-23a/p53 regulatory axis in HCC (Wang N. et al., 2013; Wang et al., 2014; Huang et al., 2015). miR-23a is part of a cluster that includes miR-27a and miR-24-2, and deregulation of the cluster or its members is associated with several cancers (Chhabra et al., 2010). miR-23a is associated with the sensitivity of ovarian cancer cells to cisplatin (Jin and Wei, 2015). miR-23a is downregulated in endometrial endometrioid adenocarcinoma and promotes EMT (Liu et al., 2016). However, miR-23a is upregulated in non-small cell lung cancer and laryngeal cancer and associated with tumor size, TNM stage, lymph node metastasis, and patient survival (Qu et al., 2015; Zhang et al., 2015). In HCC, miR-23a upregulation was identified as a risk factor for overall and recurrence free survival together with vascular invasion and tumor size, and miR-23a was found to be upregulated in SMMC-7721 and HepG2 cells (Bao et al., 2014). This may contradict the present findings, as the upregulation of TRIB1 in HCC would imply decreased levels of miR-23a. In addition, the upregulation of TRIB1 in HCC resulted in the downregulation of p53, which would further decrease miR-23a levels. The expression of miR-23a in the HCC tumor tissues and cell lines used in the present study needs to be investigated in a future study.

The results of the present study suggest a regulatory mechanism in HCC by which the downregulation of miR-23a leads to the upregulation of TRIB1. High levels of TRIB1 inhibit the tumor suppressor p53, leading to increased tumor growth and further downregulation of miR-23a. In addition, TRIB1 upregulation activates β-catenin and its effectors, promoting EMT and tumor metastasis. These findings suggest potential targets for the treatment of HCC and therefore merit further investigation. In conclusion, we have formed a new feedback loop in which TRIB1 could downregulate the miR-23a expression by inhibiting the tumor suppressor p53. Thus, our research mainly provides a meaningful method for using the TRIB1 protein as an attractive target for the HCC therapy.

## Ethics statement

This study was carried out in accordance with the recommendations of the National Institute of Health Guide for the Care and Use of Laboratory Animals and Animal Care and Use Committee at the the Seventh People's Hospital of Shanghai University of TCM. Seventh People's Hospital of Shanghai with written informed consent from all subjects. All subjects gave written informed consent in accordance with the Declaration of Helsinki.

## Author contributions

YY, GaW, GoW, and SH performed the research; JZ analyzed study data; YS provided of study materials, reagents, materials, patients, laboratory samples, animals; JN analyzed the data; JW and WX designed the research study and WX wrote the paper.

### Conflict of interest statement

The authors declare that the research was conducted in the absence of any commercial or financial relationships that could be construed as a potential conflict of interest.

## Supplementary material

Supplementary Figure 1 **(A)** Expression levels of pri-miR23a and miR-18a in HepG2 cells transfected with different p53 expression constructs as indicated, which were compared with those of the control group transfected with vector (group 1), which was set as 1. **(B)** The expression of p53 proteins in each transfection was analyzed by Western blotting as shown at the bottom of each group (*n* = 3). ^*^*p* < 0.05, ^**^*p* < 0.01 compared with the vector group.
