# Supplementary figures and images for: The Oncogenic Role of Tribbles 1 in Hepatocellular Carcinoma Is Mediated by a Feedback Loop Involving microRNA-23a and p53
Source: Front Physiol. 2017 Nov 10;8:789. doi: 10.3389/fphys.2017.00789 (PMC5686088; doi:10.3389/fphys.2017.00789)

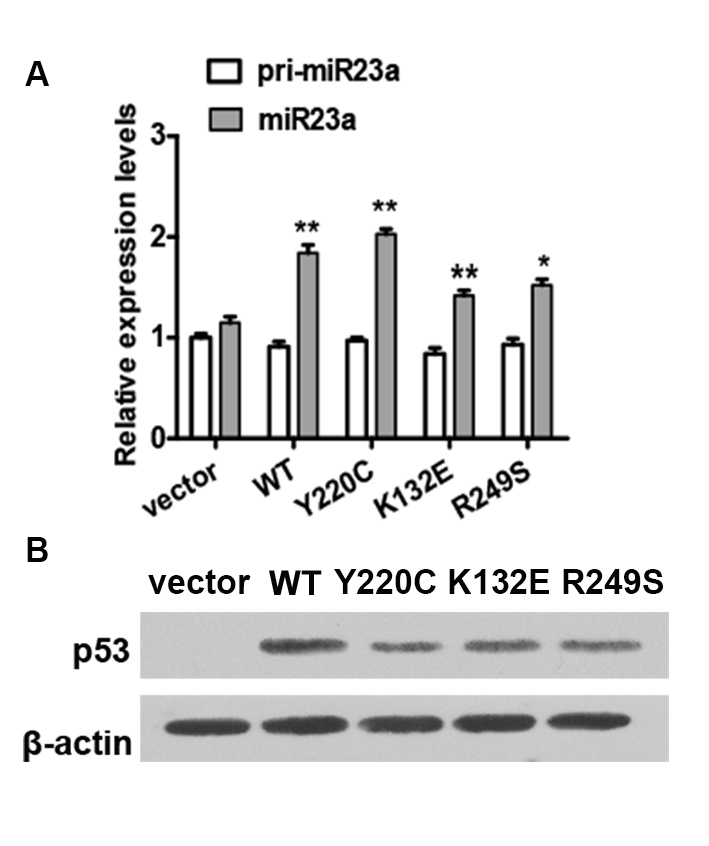

Supplement: Supplementary Figure 1 — (A) Expression levels of pri-miR23a and miR-18a in HepG2 cells transfected with different p53 expression constructs as indicated, which were compared with those of the control group transfected with vector (group 1), which was set as 1. (B) The expression of p53 proteins in each transfection was analyzed by Western blotting as shown at the bottom of each group (n = 3). *p < 0.05, **p < 0.01 compared with the vector group. [file Image1.tif]
